# Acute Region-Specific Effects of Static and Dynamic Stretching on Gastrocnemius Medialis Shear Modulus Assessed by Shear-Wave Elastography

**DOI:** 10.3390/s26216971

**Published:** 2026-11-04

**Authors:** Stefano Longo, Stefano Pau, Riccardo Battioli, Nicholas Toninelli, Giuseppe Coratella, Fabio Esposito, Luca Maria Sconfienza, Emiliano Cè

**Affiliations:** 1 Department of Biomedical Sciences for Health, Università degli Studi di Milano, Via G. Colombo 71, 20133 Milan, Italy; s.pau23@phd.uniss.it (S.P.); riccardo.battioli@unimi.it (R.B.); nicholas.toninelli@unimi.it (N.T.); giuseppe.coratella@unimi.it (G.C.); fabio.esposito@unimi.it (F.E.); luca.sconfienza@unimi.it (L.M.S.); emiliano.ce@unimi.it (E.C.); 2 Department of Biomedical Sciences, Università degli Studi di Sassari, Viale San Pietro, 43b, 07100 Sassari, Italy; 3 IRCCS Istituto Ortopedico Galeazzi–Sant’Ambrogio, Via C. Belgioioso 173, 20157 Milan, Italy

**Keywords:** muscle stiffness, passive mechanical properties, myotendinous junction, range of motion, passive resistive torque, muscle–tendon complex, muscle architecture, flexibility

## Abstract

**Highlights:**

**What are the main findings?**

- Passive stretching of different durations and dynamic stretching reduced the mid-belly shear *modulus* of the gastrocnemius medialis (GM) muscle when evaluated in a lengthened position (i.e., 80% of maximal dorsiflexion range of motion), while no changes were detected at neutral ankle angle.
- Close to the myotendinous junction, the GM shear *modulus* decreased after both passive stretching of longer duration and dynamic stretching at both ankle positions, while passive stretching of shorter duration did not affect the GM passive mechanical properties of this region.

**What are the implications of the main findings?**

- Regional shear-wave elastography can reveal stretch-induced mechanical changes that are not captured by joint-level measurements such as ROM or passive torque, highlighting the importance of measurement location and muscle length when assessing acute stretching responses.
- Stretching duration and the loading pattern may influence the mechanical response of distal muscle regions, suggesting that stretching protocols should be selected according to the specific tissue response sought rather than assuming a uniform effect throughout the muscle–tendon complex.

**Abstract:**

Stretching acutely modifies muscle–tendon mechanical behavior. Whether these effects vary across muscle regions and stretching modalities remains unclear. This study investigated the acute effects of passive static stretching (PS) of different durations and dynamic stretching (DS) on the regional gastrocnemius medialis (GM) shear *modulus*. Twelve healthy adults (4 women; mean ± SD: age = 23.9 ± 2.4 years, body mass = 69.8 ± 10.6 kg, height = 1.72 ± 0.08 m) completed three randomized conditions: 5 × 30 s PS (PS30), 5 × 45 s PS (PS45), and 5 × 30 repetitions of DS. Shear-wave elastography assessed the shear *modulus* at mid-belly (GM_mid_) and towards the myotendinous junction (GM_MTJ_) at 0° and 80% of maximal dorsiflexion range of motion (ROM_max_). ROM_max_, passive resistive torque (PRT), myotendinous junction displacement (ΔMTJ), muscle architecture, and maximal voluntary contraction (MVC) were also evaluated. The GM_mid_ *modulus* decreased at 80%ROM_max_ after all stretching protocols (*p* < 0.05), with no changes at 0°. At GM_MTJ_, the *modulus* decreased after PS45 and DS at both ankle positions (*p* < 0.05); no significant changes occurred after PS30. After all protocols, ROM_max_ increased (*p* < 0.05), while PRT and MVC decreased (*p* < 0.05), ΔMTJ increased post PS45 (*p* < 0.05), and muscle architecture remained unchanged. These findings indicate that stretching-induced changes in GM mechanical behavior can be region and muscle-length dependent, while the distal response may also be influenced by stretching duration and type.

## 1. Introduction

Stretching is widely used in sport, rehabilitation, and clinical practice to acutely increase joint range of motion (ROM) and modify the passive mechanical behavior of the muscle–tendon complex (MTC). Both passive static stretching (PS) and dynamic stretching (DS) can acutely increase ROM provided an adequate stimulus is used [1,2,3,4,5]. However, apparently similar improvements in flexibility may arise from different underlying mechanisms, including changes in stretch tolerance and alterations in the passive mechanical properties of the tissues crossing the joint [1,2,6]. Traditionally, the mechanical effects of stretching have been investigated from passive joint torque–angle relationships. However, passive torque reflects the combined resistance of several muscular and non-muscular structures and therefore cannot identify changes occurring within an individual muscle or muscle region [7,8,9]. This distinction is particularly relevant for the plantarflexors, where muscles with different mechanical properties and slack lengths contribute simultaneously to passive ankle resistance [8,9].

Shear-wave elastography (SWE) enables the non-invasive, quantitative assessment of local skeletal muscle mechanical properties by measuring the propagation velocity of mechanically induced shear waves within the tissue [10,11]. Because shear-wave propagation is related to tissue resistance to deformation, the SWE-derived shear *modulus* can be used as an index of local passive muscle stiffness under controlled conditions [8,9,12,13,14]. Unlike joint-level measurements, SWE can therefore allow specific muscles and regions to be examined independently. Thus, this technique has been used to assess passive muscle mechanical properties during and after stretching maneuvers [12,15,16]. Furthermore, previous SWE studies reported acute reductions in the gastrocnemius medialis (GM) shear *modulus* following PS [8,9,17,18] in a resting position (i.e., neutral ankle angle) [8,18] and in dorsiflexion [19]. However, skeletal muscle cannot necessarily be considered mechanically homogeneous along its length [16,19,20]. Indeed, SWE mapping during passive dorsiflexion has demonstrated regional differences in the GM shear *modulus*, with the distal region exhibiting a greater *modulus* than more proximal regions under some testing conditions despite broadly similar regional slack angles [16,19]. Such heterogeneity may reflect differences in local architecture, cross-sectional area, stress distribution, extracellular matrix organization, and force transmission along the muscle [16,19,20]. Nevertheless, most studies investigating stretch-induced changes in the GM shear *modulus* have sampled a single muscle-belly location, and little is known about whether different regions of the same GM would respond similarly to an acute stretching stimulus.

The characteristics of the stretching stimulus may further influence these local responses. For PS, changes in passive mechanical properties have been reported to depend on the duration and magnitude of the applied stimulus, suggesting that sufficient cumulative loading may be necessary to produce detectable changes in the muscle shear *modulus* [21]. DS or cyclic stretching may impose a different mechanical history, consistent with repeated stretch and recovery rather than sustained stretch at a relatively fixed muscle length. Therefore, the effects of DS on MTC mechanical behavior seem less consistent than that of PS [6,22,23,24]. Increased ankle ROM following DS has been reported both with and without detectable changes in passive torque, MTC stiffness, or muscle architecture [6,22,23]. However, overall direct comparisons between PS and DS are limited, and it remains unclear whether differences in stretching duration and loading pattern could translate into different responses across regions of the same muscle.

Therefore, the aim of the present study was to compare the acute effects of three stretching protocols (two PS of different durations and DS) on the regional GM shear *modulus*. To provide a broader characterization of the acute response, changes in maximal ROM, passive resistive torque, myotendinous junction displacement, GM architecture, and maximal voluntary contraction were also assessed. We hypothesized that stretching would reduce the GM shear *modulus*, with changes being more evident at the lengthened ankle position, and that the response would differ between the center and the distal part of the GM.

## 2. Materials and Methods

### 2.1. Participants

A priori power analysis was conducted using G*Power software (v3.1, Düsseldorf, Germany), applying a repeated-measures analysis of variance model (ANOVA-RM) to detect changes in ROM, with an expected medium effect size (f = 0.25). Assuming a significance level of α = 0.05 and statistical power (1 − β) = 0.80, the minimum required sample size was estimated at 11 participants. ROM was selected as the primary outcome for sample-size planning because the initial objective was to assess acute improvements in flexibility following practically applicable stretching protocols and their effects on the shear *modulus*. Therefore, 12 participants (four women) were recruited to account for potential dropouts. Further recruitment was limited by the availability of the SWE-equipped ultrasound system; hence, no separate sample-size calculation was performed for the regional shear *modulus*. Since menstrual cycle-related hormonal fluctuations seem to have minimal influence on muscle–tendon mechanical properties in vivo [25], the menstrual cycle phase was not controlled in women. Additionally, as sex does not appear to affect stretching-induced changes in muscle–tendon stiffness and range of motion [26], both men and women were included. Participant characteristics were as follows (mean ± SD): age = 23.9 ± 2.4 years, body mass = 69.8 ± 10.6 kg, and height = 1.72 ± 0.08 m. All volunteers were physically active university students who engaged in recreational sports activities (2.0 ± 1.0 h/week). Prior to inclusion, participants received a detailed explanation of the study aims and procedures, and each provided written informed consent. Exclusion criteria included (i) musculoskeletal injury to the lower limbs within the previous six months; (ii) neurological conditions impairing flexibility; (iii) regular participation in structured stretching programs; and (iv) any contraindication to stretching exercises (e.g., joint hypermobility, connective-tissue disorders). Participants were instructed to refrain from consuming ergogenic aids or similar substances during the 24 h preceding testing and to avoid strenuous physical activity in the 48 h prior to each experimental session. The study protocol received ethical approval from the University Ethics Committee (CE84/23) and was conducted in accordance with the Declaration of Helsinki.

### 2.2. Experimental Design

A randomized, within-subject crossover design was employed to investigate the acute effects of different stretching modalities on the regional GM shear *modulus*. Each participant first completed a familiarization session to become accustomed to the experimental procedures and stretching protocols. Subsequently, participants attended three experimental sessions, each corresponding to a specific stretching condition: (i) passive static stretching of 5 × 30 s (PS30); (ii) passive static stretching of 5 × 45 s (PS45); and (iii) dynamic stretching of 5 × 30 repetitions (DS). The order of the sessions was randomized, and a minimum interval of 72 h was maintained between sessions (Figure 1). Protocol details are provided below.

All experiments were conducted in a temperature-controlled environment (22 ± 1 °C) with relative humidity (50 ± 5%). During assessments, participants were positioned prone on a custom-built dynamometer [27], consisting of a medical examination bed equipped with a movable metal plate connected to a previously calibrated load cell (model SM-2000 N, Interface, Crowthorne, UK), operating linearly (range: 0–2000 N). The ankle of the dominant leg was securely fixed to the movable plate using a Velcro^®^ strap to prevent heel displacement during testing. The hips and shoulders were also stabilized. The load cell was aligned with the direction of force application throughout all measurements. The force signal was transmitted to an analog-to-digital converter (model UM150, Biopac Systems Inc., Santa Barbara, CA, USA), sampled at 1 kHz, and stored on a personal computer for subsequent analysis. Joint torque was calculated by multiplying the measured force by the external lever arm of the dynamometer. This was measured along the footplate as the distance between the projection of the ankle joint rotation center, identified using the lateral malleolus as an anatomical reference, and the fixed load-cell location, using a tape measure with 1 mm graduations. With the foot secured to the footplate, this distance remained constant throughout the assessments. The ankle joint angle was continuously monitored using a calibrated biaxial electrogoniometer (model TSD130A, Biopac Systems Inc., CA, USA) positioned along the lateral aspect of the fibula and the calcaneus.

Each session followed the same experimental sequence. After ankle fixation, participants performed a standardized warm-up consisting of 10 passive ankle movements of increasing intensity administered by an operator and 10 active ankle movements. This was followed by baseline measurements (PRE). Maximal dorsiflexion ROM (ROM_max_) was first determined. The ankle joint was then positioned at two angles in randomized order: 0° (defined as the perpendicular alignment between the tibia and the longitudinal axis of the foot) and 80% of each individual ROM_max_ (80%ROM_max_). This position was chosen to minimize potential saturation of the SWE signal, especially at the myotendinous junction (MTJ), and to ensure reliable measurements by maintaining the reliability index ≥0.4 (see Section 2.4). Furthermore, this angle lies beyond the slack length of the MTC, where passive tension is present, and it has been shown to be sensitive to stretch-induced changes in both the GM shear *modulus* and passive resistive torque (PRT) [8].

At both positions, ultrasound images of the GM were acquired, and PRT, as an index of stretch tolerance, was measured at 80%ROM_max_. Of note, POST-intervention PRT was recorded at the same absolute angle determined at PRE. Subsequently, maximal voluntary isometric contraction (MVC) of the plantarflexors was assessed at 0°. Participants were then released from the dynamometer and performed the assigned stretching protocol in a standing position on a wedge platform. Myoelectric activity was monitored throughout the intervention to detect potential reflex muscle activation. Immediately after the intervention (POST), participants were repositioned on the dynamometer, and all measurements were repeated under the same conditions.

Besides checking for potential differences among days at PRE for all variables, a control condition (CTRL) for ROM_max_ and SWE image acquisition was performed at the beginning of each session to assess the efficacy of each stretching intervention on the main outcomes. In this condition, ROM_max_ and SWE images were collected PRE and POST a 5 min rest period off the dynamometer, thus matching the duration of the interventions. Following CTRL, a 10 min rest period was provided before the start of the experimental intervention (Figure 1).

### 2.3. ROM_max_ and PRT Assessment

Ankle joint ROM_max_ was determined starting with the ankle at its resting position (~−20° of dorsiflexion) and then manually dorsiflexed at slow speed by an external operator to avoid reflex activations monitored by surface electromyography (sEMG) until the participant’s point of discomfort was reached [27]. Prior to sEMG electrode placement, the skin was prepared to reduce impedance at the electrode–skin interface. The areas over the muscles of interest were shaved, gently abraded using an abrasive gel (Nuprep, Weaver and Co., Aurora, CO, USA), and cleaned. sEMG signals were recorded using bipolar Ag/AgCl electrodes (active area: 1 cm^2^; ARBO Kendall, Covidien LLC, Mansfield, MA, USA) with a 20 mm interelectrode distance. Signals were acquired at a sampling frequency of 1 kHz using a wireless system (Free-EMG, BTS Bioengineering, Garbagnate Milanese, Italy). Electrodes were placed over the muscle bellies of the gastrocnemius lateralis and soleus in accordance with established recommendations [28]. The gastrocnemius lateralis was selected instead of the GM to avoid interference with ultrasound measurements performed on the latter. To verify correct electrode placement and signal quality, participants were asked to perform brief voluntary contractions of the target muscles while the sEMG signal was visually inspected by an experienced operator [29]. sEMG data were collected solely to monitor potential reflex activity; hence, these data were not recorded nor included in the subsequent analysis.

The difference between the ankle at 0° and the end ROM was considered as ROM_max_. Reliability was previously reported (intraclass correlation coefficient − ICC = 0.94; standard error of measurement as percentage − SEM% = 1.1) [30].

Following ROM_max_ assessment, ultrasound measurements were performed at 0° and 80%ROM_max_. At the latter position, PRT of the plantarflexors was recorded as the mean force over the first 5 s following ankle fixation [27]. Such a short time allowed the operator to minimize the influence of the static position on the MTC viscoelastic properties.

### 2.4. Ultrasound Measurements

Ultrasound measurements (i.e., SWE, muscle architecture and MTJ displacement) were performed in vivo at rest by an experienced operator (≥10 years of experience) using a B-mode ultrasound system (V8, Samsung Healthcare, Seoul, Republic of Korea) equipped with a 5 cm linear-array transducer (model LA2-14A, 2.0–14.0 MHz, Samsung Healthcare, Seoul, Republic of Korea). Participants lay prone on an examination bed with the hip and knee fully extended and the ankle fixed at 0°. Acoustic gel was applied to ensure optimal coupling. The proximal and distal ends of the GM were identified by moving the transducer along the longitudinal axis of the muscle belly. The MTJ was then identified and marked on the skin using hypoechoic tape, serving as a reference for displacement analysis (see below) [27,31,32]. Subsequently, the midpoint of the muscle belly (50% of its length) was marked on the skin. At this site, the medial and lateral borders of the muscle were identified, and the midpoint of the muscle width was also marked. The transducer was positioned longitudinally to obtain images including both superficial and deep aponeuroses. The probe orientation was adjusted to clearly visualize the muscle fascicles running between the aponeuroses and that the region of interest for the SWE measurements was free from blood vessels and muscle fascia [33]. To ensure consistent probe placement across measurements, the transducer location at both the center of the muscle belly (GM_mid_) and MTJ (GM_MTJ_) was outlined on the skin using a dermographic pencil. Care was taken to minimize probe pressure throughout all subsequent acquisitions.

Once the anatomical locations were identified, the ultrasound system was switched to S-Shearwave Imaging™ mode. The ankle was positioned at both 0° and 80%ROM_max_ to acquire images at GM_mid_ and GM_MTJ_. The order of ankle positions was randomized, as was the order of measurement sites within each angle. A generous amount of transmission gel was applied to ensure optimal acoustic coupling. A rectangular region of interest (ROI; 3 cm × 2.5 cm) displaying a color-coded elasticity map was visualized. During probe placement, particular attention was paid to orient the transducer so that fascicles were clearly visible at both ankle angles concomitantly, avoiding large vascular structures. This minimized possible between-angle differences in the *modulus* due to changes in muscle architecture per se [12]. Alongside the elasticity map, a second image displaying the reliability measure index (RMI) was automatically provided by the system (Figure 2). The RMI ranged from 0 to 1.0, with values ≥0.4 considered acceptable [34,35].

For each condition, three images were acquired at each ankle angle and site, stored, and subsequently analyzed offline. At GM_mid_, two circular ROIs (1 cm in diameter) were placed within the muscle belly, avoiding the superficial and deep aponeuroses and preferentially selecting areas with the highest RMI. The *modulus* within each ROI was automatically calculated by the system and expressed in kPa. The two values were averaged for each image, and the mean of the three images was then computed to obtain a representative *modulus* value for GM_mid_ at each angle. The same procedure was applied to GM_MTJ_ images, with the only difference that three smaller ROIs were positioned within this region (Figure 2).

GM muscle architecture was assessed using the same ultrasound images acquired for SWE at GM_mid_, at both 0° and 80%ROM_max_. Image analysis was performed offline using open-source software (ImageJ 1.44b, National Institutes of Health, Bethesda, MD, USA). For each image, three distinct and clearly identifiable fascicles were selected. Fascicle length (L_f_) was determined by tracing each fascicle between the superficial and deep aponeuroses, accounting for any visible curvature. The fascicle angle (θ) was defined as the angle formed between each measured fascicle and the deep aponeurosis. The three measured fascicles and angles were averaged. Muscle thickness (MT) was calculated as the distance between the superficial and deep aponeuroses, measured at three different locations and subsequently averaged. All architectural variables were measured from three images acquired at each joint angle (PRE and POST interventions), and the corresponding values were averaged prior to statistical analysis.

The position of the MTJ was visualized in a continuous sagittal plane at 0° and 80%ROM_max_. The MTJ displacement at 80%ROM_max_ (ΔMTJ) relative to the hypoechoic marker was captured by ultrasound PRE and POST interventions and digitized offline with ImageJ. As previously described [31], possible artifacts due to skin shift during MTJ displacement assessment were corrected accordingly [27,32]. ΔMTJ was used as an index of muscle elongation.

### 2.5. MVC Assessment

Following a standardized warm-up (10 × 2 s contractions of increasing intensity from 50% MVC determined during familiarization up to maximum), the plantarflexor MVC was evaluated at 0°. Participants were instructed to perform each contraction “as fast and hard as possible”, sustain the effort for ~3 s, and then fully relax. Two trials were completed, with a 2 min recovery period between attempts. If the difference between trials exceeded 5%, an additional trial was performed. The highest value obtained was retained for subsequent analysis. As MVC was not a primary outcome of the study, it was assessed last in the testing sequence at both PRE and POST to minimize potential interference with the other measurements.

### 2.6. Stretching Interventions

All stretching protocols targeted the plantarflexor muscle group of the dominant limb and were performed in a standardized standing position on a custom wedge platform to induce ankle dorsiflexion. The standing wedge exercise was selected to reflect a practical stretching setting in which intensity is self-regulated according to perceived discomfort. Participants stood facing a fixed support structure, with both hands placed on a horizontal bar at approximately chest height to ensure balance and minimize upper-body involvement (Figure 3). The tested limb was positioned anteriorly with the knee fully extended, while the contralateral limb was placed posteriorly for stability. Participants were instructed to maintain an upright trunk and avoid compensatory movements at the hip or knee throughout all conditions. This exercise was selected, as it represents a stretching modality commonly used in recreational and sport settings. Each session was supervised by an experienced operator, who ensured correct exercise execution, controlled the timing of stretching and rest intervals using a metronome, and verified the absence of reflex muscle activation via sEMG monitoring. Participants practiced the exercise and identified their maximal tolerable stretch-related discomfort during familiarization; this criterion was subsequently applied across all protocols.

For PS30 and PS45, ankle dorsiflexion was achieved by leaning against the support bar until a maximally tolerable stretch within the pain limit was reached [27,36]. In PS30, 5 × 30 s bouts were performed, each separated by 30 s of rest (total stretching volume = 150 s). In PS45, 5 × 45 s bouts were performed with 15 s of rest between bouts (total stretching volume = 225 s) [27]. During each bout, participants were instructed to remain relaxed and avoid any voluntary muscle contraction. The inclusion of both PS30 and PS45 allowed the investigation of a potential dose–response relationship between time under stretch and potential regional changes in the shear *modulus*. PS45 was deliberately included to examine the response to a longer static stretching exposure, increasing cumulative time at the end ROM from 150 to 225 s while maintaining five bouts. Rest intervals were shortened from 30 to 15 s to retain the same overall protocol duration.

During DS, participants performed 5 × 30 controlled repetitions. Each repetition consisted of a forward movement achieved by flexing the elbows while leaning against the support bar to reach maximal ankle dorsiflexion within 4 s (stretching volume = 150 s at maximal dorsiflexion), followed by a return to the starting position through elbow extension over 4 s. Movement cadence was standardized using a metronome. A 30 s rest interval was provided between sets.

### 2.7. Statistical Analysis

Statistical analysis was performed using a statistical software package (IBM SPSS Statistics v. 30, Armonk, NY, USA). The Shapiro–Wilk test was used to check the normal distribution of the sampling. Sphericity was assessed using Mauchly’s test, and the Greenhouse–Geisser correction was applied when this assumption was violated. To determine possible baseline differences at PRE, a one-way analysis of variance for repeated measures (ANOVA-RM) was applied for each variable of interest. Each day, a 2 × 2 ANOVA-RM (within-factor *time*: PRE and POST; within-factor *condition*: CTRL and stretching) was used to assess the effects of each type of stretching intervention compared to its respective control condition for ROM_max_ and SWE-derived measures. A 2 × 3 ANOVA-RM (*time*: PRE and POST; *intervention*: PS30, PS45, and DS) was applied to check for the effects of the stretching interventions on all dependent variables. A 2 × 2 ANOVA-RM (*time*: PRE and POST; *angle*: 0° and 80%ROM_max_) was used to assess the potential stretch-induced effects on muscle architecture variables and the interaction with ankle angle. When the variables were not normally distributed, the corresponding non-parametric tests for repeated measures were applied (Friedman and Wilcoxon tests). In case of significant main effects or interactions, pairwise comparisons were carried out with Bonferroni correction. The ANOVA effect size was evaluated with partial eta squared (η_p_^2^) and classified as follows: <0.06: *small*; if, 0.06–0.14: *medium*; and >0.14: *large* [37]. The shear *modulus* ICC (two-way mixed, absolute agreement) with its 95% confidence interval (95%CI) was calculated intra-day for each session. ICCs were classified as follows: ICC < 0.5, poor; 0.5 ≤ ICC ≤ 0.75, moderate; 0.76 ≤ ICC ≤ 0.90, good; ICC>0.90, excellent [38]. The standard error of measurement in percentage (SEM%) and the minimum detectable change at 95% confidence (MDC95) were also calculated [39]. Data are presented in mean ± SD. Statistical significance was set at *p* < 0.05.

## 3. Results

### 3.1. Shear Modulus Reliability

Intra-session reliability, assessed using PRE and POST CTRL measurements, yielded ICCs ranging from 0.846 to 0.994, with SEM values of 2.17–4.68% and MDC95 values of 6.01–12.98% (Table 1).

### 3.2. ROM_max_

The results of the ANOVA-RM for the control analysis are reported in Table 2. No significant differences were observed between the PRE stretching condition and its corresponding PRE CTRL (*p*-range = 0.08–0.97). When comparing each stretching condition with its corresponding control, significant main effects of *time* (*p* < 0.05), *condition* (*p* < 0.05), and interaction (*p* < 0.01) were consistently observed. In CTRL of PS30, PS45, and DS, ROM_max_ did not change significantly between PRE and POST (*p*-range = 0.16–0.79). In contrast, ROM_max_ increased significantly following all stretching interventions by 8.2% POST PS30 (*p* = 0.001), 7.8% POST PS45 (*p* < 0.001), and 10.2% POST DS (*p* < 0.001). Comparing only the three stretching interventions, no significant differences were found at PRE (F = 1.21, *p* = 0.32, η_p_^2^ = 0.10). The ANOVA-RM revealed a main effect of *time* (F = 74.23, *p* < 0.001, η_p_^2^ = 0.87), while no significant effect of *intervention* (F = 1.74, *p* = 0.20, η_p_^2^ = 0.14) or interaction (F = 0.52, *p* = 0.60, η_p_^2^ = 0.05) was found, indicating similar stretch-induced increases in ROM_max_ by the different stretching stimuli.

### 3.3. PRT at 80%ROM_max_

Comparing the three stretching interventions, no significant differences were found at PRE (F = 0.95, *p* = 0.95, η_p_^2^ = 0.005). The ANOVA-RM revealed only a main effect of *time* (F = 31.44, *p* < 0.001, η_p_^2^ = 0.74), while no significant effect of *intervention* (F = 0.07, *p* = 0.93, η_p_^2^ = 0.01) or interaction (F = 0.17, *p* = 0.84, η_p_^2^ = 0.02) was found, indicating similar stretch-induced decrements in PRT by the different stretching stimuli. PRT at 80%ROM_max_ decreased significantly by 14.2% in PS30 (*p* = 0.004), 12.1% in PS45 (*p* = 0.009), and 10.3% in DS (*p* = 0.001) (Figure 4A).

### 3.4. GM_mid_ Modulus

The results for the control analysis are reported in Table 2. No significant differences were observed between each PRE stretching condition and its corresponding PRE CTRL (*p*-range = 0.43–0.94).

At 0°, the Friedman test did not disclose significant differences when comparing each stretching condition with its corresponding control (*p*-range = 0.06–0.38), indicating that at this ankle angle, none of the stretching stimuli induced significant changes in the *modulus* (Figure 5A).

At 80%ROM_max_, the ANOVA-RM always revealed significant main effects of *time* (*p* < 0.05), *condition* (*p* ≤ 0.01), and interaction (*p* < 0.01) when comparing each stretching condition with its corresponding control (Table 2). In CTRL of PS30, PS45, and DS, the *modulus* did not change significantly between PRE and POST (*p*-range = 0.43–0.60). In contrast, the *modulus* decreased significantly following all stretching interventions (*p* < 0.01). Comparing the three stretching interventions, no significant differences were found at PRE (F = 0.33, *p* = 0.64, η_p_^2^ = 0.03). The ANOVA-RM revealed only a main effect of *time* (F = 44.38, *p* < 0.001, η_p_^2^ = 0.80), while no significant effect of *intervention* (F = 0.01, *p* = 0.99, η_p_^2^ = 0.001) or interaction (F = 0.62, *p* = 0.54, η_p_^2^ = 0.05) was found, indicating similar stretch-induced decrements in the *modulus* by the different stretching stimuli. Percentage changes are shown in Figure 5B.

### 3.5. GM_MTJ_ Modulus

The results for the control analysis are reported in Table 2. No significant differences were observed between each PRE stretching condition and its corresponding PRE CTRL (*p*-range = 0.14–0.62).

At 0°, when comparing PS30 with its corresponding CTRL, the ANOVA-RM did not disclose significant differences (*p* > 0.05). When comparing PS45 with its corresponding CTRL, only a significant main effect of *time* (*p* < 0.01) was found. Pairwise comparisons revealed that in CTRL, the *modulus* did not change significantly between PRE and POST (*p* = 0.66), while the *modulus* decreased significantly after PS45 (*p* = 0.04). Comparing DS with its CTRL, the analysis showed main effects of *time* (*p* = 0.001) and interaction (*p* = 0.001), although no main *condition* effect was retrieved (*p* = 0.09). Pairwise comparisons highlighted that the *modulus* did not change significantly between PRE and POST in CTRL (*p* = 0.70), while the *modulus* decreased significantly POST DS (*p* < 0.001). Comparing the three stretching interventions, no significant differences were found at PRE (F = 0.49, *p* = 0.53, η_p_^2^ = 0.04). The ANOVA-RM revealed only a main effect of *time* (F = 19.10, *p* = 0.001, η_p_^2^ = 0.63), while no significant effect of *intervention* (F = 1.00, *p* = 0.38, η_p_^2^ = 0.08) or interaction (F = 0.80, *p* = 0.46, η_p_^2^ = 0.07) was found. After stretching, the *modulus* decreased significantly only in PS45 (*p* = 0.04) and DS (*p* < 0.001). Percentage changes are shown in Figure 5C.

At 80%ROM_max_, the Friedman test did not highlight significant differences when comparing PS30 with its CTRL (*p* = 0.44), indicating no stretch-induced changes in the *modulus* at this ankle angle. In PS45, the ANOVA-RM revealed significant main effects of *time* (*p* < 0.01) and interaction (*p* < 0.05), while significant main effects of *time* (*p* < 0.01) and *condition* (*p* < 0.05) without significant interaction (*p* > 0.05) were retrieved in DS. Pairwise comparisons showed that the *modulus* did not change significantly between PRE and POST CTRL in either PS45 (*p* = 0.66) or DS (*p* = 0.70). In contrast, the *modulus* decreased significantly POST PS45 (*p* = 0.003) and DS (*p* = 0.03). Comparing the three stretching interventions, no significant differences were found at PRE (F = 0.49, *p* = 0.62, η_p_^2^ = 0.04). The ANOVA-RM revealed only a main effect of *time* (F = 13.55, *p* = 0.004, η_p_^2^ = 0.55), while no significant effect of *intervention* (F = 1.50, *p* = 0.25, η_p_^2^ = 0.12) or interaction (F = 2.63, *p* = 0.09, η_p_^2^ = 0.19) was found. After stretching, the *modulus* decreased significantly only in PS45 (*p* = 0.003) and DS (*p* = 0.03) Percentage changes are shown in Figure 5D.

### 3.6. Muscle Architecture

The architectural parameters are shown in Table 3. No significant differences were found at PRE among interventions in any architectural parameter at either 0° (L_f_: F = 0.53, *p* = 0.59, η_p_^2^ = 0.05; θ: F = 0.35, *p* = 0.71, η_p_^2^ = 0.03; MT: F = 1.08, *p* = 0.36, η_p_^2^ = 0.09) or 80%ROM_max_ (L_f_: F = 3.20, *p* = 0.06, η_p_^2^ = 0.23; θ: F = 0.14, *p* = 0.87, η_p_^2^ = 0.01; MT: F = 2.21, *p* = 0.16, η_p_^2^ = 0.17). The ANOVA-RM revealed that within each stretching intervention, only a main effect of *angle* was found in all variables (*p* < 0.01), while no effects of *time* (*p* > 0.05) or interaction (*p* > 0.05) were highlighted. Pairwise comparisons showed that L_f_ was always significantly longer at 80%ROM_max_ than at 0° (*p* < 0.01), θ was smaller and MT was greater at 80%ROM_max_ than at 0° (*p* < 0.01) (Table 3).

### 3.7. Muscle Elongation

ΔMTJ is shown in Figure 4B. No significant differences were found at PRE among the interventions (F = 0.05, *p* = 0.96, η_p_^2^ = 0.01). The ANOVA-RM revealed only a significant main effect of *time* (F = 12.26, *p* = 0.005, η_p_^2^ = 0.53), without a main effect for *intervention* (F = 0.13, *p* = 0.88, η_p_^2^ = 0.01) or interaction (F = 1.28, *p* = 0.30, η_p_^2^ = 0.10). Pairwise comparisons showed that after stretching, ΔMTJ was significantly greater only in PS45 (27.5%, *p* = 0.02), whereas increments in PS30 (16.9%) and DS (6.1%) were not statistically significant (*p* = 0.10 and 0.32, respectively).

### 3.8. MVC

MVC is shown in Figure 4C. No significant differences were found at PRE among the interventions (F = 0.08, *p* = 0.92, η_p_^2^ = 0.01). The ANOVA-RM revealed only a significant main effect of *time* (F = 25.17, *p* < 0.001, η_p_^2^ = 0.70), without a main effect for *intervention* (F = 0.16, *p* = 0.85, η_p_^2^ = 0.01) or interaction (F = 0.25, *p* = 0.78, η_p_^2^ = 0.02). Pairwise comparisons showed that MVC significantly decreased by 9.4% POST PS30 (*p* = 0.003), by 11.1% POST PS45 (*p* < 0.001), and by 9.3% POST DS (*p* = 0.02).

## 4. Discussion

The main aim of this study was to use SWE to compare the acute, region-specific effects of three commonly used stretching protocols on the GM shear *modulus* as an index of stiffness. The main finding was a different stretch-induced response between the GM_mid_ and GM_MTJ_ locations, which further depended on ankle joint position. At the muscle belly, the three stretching modalities yielded similar outcomes, such as a reduced shear *modulus* at the lengthened position, with no differences among interventions. In contrast, modality and duration mattered at GM_MTJ_, with PS30 not altering the local shear *modulus* at either ankle angle, whereas PS45 and DS had a decreased *modulus* at both joint angles. This regional difference was also accompanied by the largest muscle elongation in PS45. Notably, all interventions increased ROM_max_ and stretch tolerance and decreased MVC without altering muscle architecture. Collectively, the findings could indicate that the proposed stretching protocols did not uniformly modify the passive mechanical behavior of the GM and highlight the importance of considering both measurement location and muscle length when interpreting acute changes in the shear *modulus*.

### 4.1. Preliminary Considerations

The inclusion of a time-matched control condition was intended to determine whether changes in ROM_max_ and the regional shear *modulus* reflected real responses to stretching rather than day-to-day variability, repeated measurements, or other time-dependent factors. The absence of PRE-to-POST changes in these variables at either GM_mid_ or GM_MTJ_ at both ankle positions supported the stability of these measurements. Moreover, the shear *modulus* exhibited good-to-excellent intra-day reliability and low SEM% (i.e., <5.0%) for almost all conditions. Importantly, all the significant differences in the *modulus* exceeded the MDC95. Therefore, the changes observed after the stretching interventions are unlikely to reflect measurement fluctuation or repeated testing alone.

The accompanying changes in ROM_max_, PRT, and ΔMTJ provide a broader mechanical context for the regional changes in the shear *modulus*. ROM_max_ increased after all three interventions, with similar improvements following PS30, PS45, and DS. These findings are consistent with previous studies using comparable stretching protocols [1,2,4,5]. Acute increases in joint ROM following stretching were generally attributed to a combination of altered stretch tolerance [36,40] and changes in the passive mechanical behavior of the tissues resisting joint motion [1,41]. In the present study, a mechanical contribution was supported by the reduction in PRT at 80%ROM_max_ after all interventions, together with an overall increase in GM elongation, although the pairwise increase reached statistical significance only after PS45. Because PRT was assessed at the same absolute ankle angle PRE and POST, the lower POST values would indicate reduced passive mechanical resistance at the same imposed joint position. As the mechanisms underlying these changes may overlap with those responsible for the observed reductions in the shear *modulus*, they are discussed in detail below.

### 4.2. Stretch-Induced Effects on GM Shear Modulus

According to the literature, the GM_mid_ shear *modulus* at 0° was very low [8,12,15,16], suggesting that although the GM was not necessarily slack at 0° [8,16], it developed very little passive tension. After stretching, no changes in the GM_mid_ shear *modulus* were observed at this angle. Contrasting with our findings, previous studies found a reduction in the GM *modulus* with the ankle in neutral position (i.e., 0°) after 5 × 1 min PS [17] and 4 × 30 s PS [18]. These discrepancies may reflect differences in ultrasound systems and processing algorithms, measurement timing, probe location and alignment, and ROI selection. In particular, in the present study, the ROIs were maximized and averaged across two adjacent measurement sites to capture a large portion of muscle tissue. Although this broader sampling may have provided a more representative estimate of the GM_mid_ region, spatial averaging could have also attenuated small or focal changes, especially at 0°, where *modulus* values and passive tissue loading were low. Moreover, the absence of a change at 0° in the present study seems to contrast with the increase in GM shear-wave speed reported by Pamboris et al. [42] after DS. However, their protocol involved repeated active plantarflexor contractions throughout a large ankle excursion, whereas the present DS was externally driven and performed without voluntary plantarflexor activation, which may account for the different response at 0°.

Increasing dorsiflexion up to 80%ROM_max_ greatly increased the GM_mid_ shear *modulus*, indicating a steep increase in passive tension at this angle, as also previously shown [8,9,15,16]. Interestingly, all stretching interventions reduced the *modulus* measured at this dorsiflexed position. This angle-dependent response suggests that acute stretch-induced alteration in the local passive mechanical behavior of the muscle belly became detectable when the muscle was assessed under great passive tension. In line with our results, Hirata et al. [8] reported that 5 min of continuous PS reduced the GM shear *modulus* during passive dorsiflexion, whereas no changes were observed in the lateral gastrocnemius or soleus. Moreover, Nakamura et al. [18] reported that 4 × 30 s PS (comparable to the present PS30) decreased both the GM shear *modulus* and muscle stiffness in a dorsiflexed position, with the rate of change in the *modulus* significantly correlating with the rate of change in muscle stiffness. Pamboris et al. [42] found that the GM shear-wave speed decreased at maximal dorsiflexion after an acute DS protocol, while Oba et al. [24] reported reduced GM shear-wave velocity after 4 × 30 s of DS.

The reduction in the GM_mid_ *modulus* after stretching likely reflected both a shift in the onset of passive tension and an alteration in the passive mechanical behavior of the muscle at long lengths, which were also supported by the present stretch-induced changes in muscle elongation and PRT, as discussed below. Indeed, previous studies observed that PS shifted the GM slack angle toward greater dorsiflexion and that accounting for this shift markedly reduced the apparent decrease in the *modulus* measured at the same ankle angle [8,9]. Moreover, Hirata et al. [9] found that the GM shear *modulus* measured pre- and post-PS at the same fascicle length remained reduced when the fascicles were substantially lengthened (i.e., dorsiflexion), indicating a true reduction in passive fascicle stiffness towards the end ROM. Several passive structures may have contributed to the present GM_mid_ length-dependent response. A thixotropic mechanism related to the short-range elastic component appears unlikely because relatively small preliminary movements seem to largely abolish this behavior [31]. Moreover, a stretch-induced increase in muscle fascicle extensibility seems unlikely since none of the present interventions caused changes in muscle architecture at either ankle position, which is also in line with previous studies [9,32]. Therefore, more plausible contributors to the stretch-induced decrease in the *modulus* could include transient changes in non-contractile proteins, such as titin, and deformation or rearrangement of the intramuscular connective-tissue network, particularly the endomysium and perimysium, which add passive resistance as the muscle is lengthened [7,8,9,18,31]. This length-dependent recruitment of passive structures may also explain why the GM_mid_ *modulus* decreased at 80%ROM_max_ and remained unchanged at 0° after stretching. Finally, because fascicle and whole-muscle elongation are not necessarily equivalent in a three-dimensional pennate muscle, unchanged L_f_ does not exclude altered deformation of the muscle belly or its surrounding intramuscular structures.

Interestingly, PS and DS may have produced similar reductions in the *modulus* through partly different mechanisms. Potentially, repeated loading and unloading during DS could reduce muscle viscosity and change titin-related passive tension [6,43]. However, because DS generally does not alter resting L_f_, fascicle extensibility or stiffness [22,23,43,44], changes at the fascicle level are unlikely to be the primary mechanism underlying the reduction in the *modulus*. Instead, DS may have reduced friction-related energy dissipation within the MTC, potentially through sliding between collagenous structures [45,46]. Evidence from previous DS studies also suggests that changes in whole-MTC deformation may occur without proportional changes in fascicle behavior. Indeed, Samukawa et al. [22] observed increased MTJ displacement despite unchanged L_f_ and θ, whereas Pamboris et al. [42] reported reduced GM fascicle strain at maximal dorsiflexion despite increased ankle ROM_max_. Although the latter study employed an active DS protocol, collectively, these findings suggest that cyclic stretching may redistribute elongation among fascicular and non-fascicular components arranged in series within the MTC [22,45]. A similar interpretation could be compatible with the non-significant increase in ΔMTJ and unchanged muscle architecture observed after DS in the present study, although the respective contributions of intramuscular connective tissues, aponeuroses, and tendons cannot be determined.

The stretch-induced response at GM_MTJ_ differed from that observed at GM_mid_. It is important to highlight that previous research reported a non-uniform distribution of the shear-wave *modulus* between the center of the muscle belly and the distal part [16,19], which was also observed in the present study. The authors proposed that the smaller cross-sectional area of the distal muscle may result in greater local stress and that passive tension may be greater in the muscle region closer to the extremity being stretched than in other muscle regions [16]. Moreover, the tissue surrounding the GM_MTJ_ differs structurally from the central muscle belly [47]. Indeed, muscle fibers approaching the MTJ terminate within a specialized extracellular matrix environment responsible for transmitting force between muscle and tendon. Furthermore, Jakobsen et al. [47] demonstrated abundant expression of several collagen types at the human MTJ, identified collagen XXII specifically at the muscle–tendon interface, and showed distinct distributions of collagen within the endomysium and perimysium surrounding fibers close to the MTJ. Hence, this area would result in a structurally specialized environment rather than simply an extension of the central muscle belly. In this context, we observed that PS30 did not modify the GM_MTJ_ shear *modulus* at either ankle angle, whereas reductions were present after PS45 and DS at both 0° and 80%ROM_max_. One possible explanation for this pattern is that the longer cumulative end-range exposure in PS45 and the repeated loading–unloading cycles in DS provided different stimuli for time-dependent changes in local passive tension or strain distribution within the distal GM. Given the specialized extracellular matrix organization of this region, these loading histories might have influenced its mechanical response differently from that of the central muscle belly. However, these mechanisms were not directly assessed. The absence of a significant change after PS30 could also reflect a smaller response relative to measurement variability and should not be strictly interpreted as evidence that this protocol had no effect. Another important point concerns the absence of a significant time × intervention interaction, which prevents interpreting this pattern as evidence of differential protocol efficacy or a duration-dependent response. Therefore, the present regional differences could be interpreted solely based on within-condition comparisons. Finally, although all protocols were performed up to maximal tolerable discomfort, this perceptual criterion was not necessarily equivalent to mechanical intensity. As joint angle and applied torque were not recorded during stretching, differences in the mechanical stimulus cannot be excluded as contributors to the observed responses. In contrast, PS30 and DS had comparable overall duration, though they differed markedly in loading pattern, with DS exposing the MTC to repeated passive loading–unloading cycles rather than sustained end-range passive loading. Thus, the similar GM_MTJ_ *modulus* response after PS45 and DS may reflect changes in local passive tension and/or the redistribution of strain among muscular and connective-tissue structures in the distal GM, and it could have occurred through different mechanical pathways. Additionally, the significant increase in ΔMTJ after PS45 could be compatible with greater longitudinal GM elongation over the same joint excursion, thus providing further support for altered MTC behavior after the static protocol of greatest volume. However, ΔMTJ did not increase significantly after DS despite reductions in the GM_MTJ_ *modulus*. Thus, changes in ΔMTJ cannot be considered the direct cause of the local *modulus* response. Rather, these observations would emphasize that whole-muscle elongation and the local tissue *modulus* represent related yet distinct aspects of MTC mechanics.

Importantly, PRT and the local shear *modulus* should not be regarded as interchangeable measures. PRT represents the combined resistance of multiple muscles together with tendon, fascia, neural and articular structures, whereas the shear *modulus* provides a localized measure within a specific muscle region [21]. Indeed, in the present study, PRT decreased after all protocols, whereas the local response differed between GM_mid_ and GM_MTJ_. Thus, the decrease in PRT supports an overall reduction in passive mechanical resistance without fully explaining the spatial distribution of the shear *modulus* changes.

### 4.3. Stretch-Induced Effects on MVC

MVC decreased similarly after all three interventions. The decreases following PS30 and PS45 were consistent with the duration-dependent impairment in maximal force reported after prolonged static stretching [1,4,48]. Stretch-induced force loss probably reflects a combination of neural and peripheral mechanisms, including transient reductions in voluntary activation and motoneuron excitability together with alterations in contractile and passive muscle properties [1,2,48,49]. We cannot separate these mechanisms from the present results; however, this was not the aim of our study. Interestingly, the decrease following DS seemed to contrast with the literature because conventional active DS generally produced neutral or slightly positive effects on subsequent performance, partly through possible contraction-induced increases in temperature, neural activation, and potentiation [1,6]. However, the present DS protocol consisted of passive cyclic stretching rather than conventional active DS and did not require forceful plantarflexor contractions. Consequently, the warming and potentiating effects typically associated with active DS were likely limited.

A further methodological consideration is that MVC was assessed after completion of the ultrasound measurements, which included periods in which the ankle was maintained at 80%ROM_max_. Although we tried to minimize the time for image acquisition, this additional passive loading at a lengthened muscle position may have reinforced or prolonged the stretch-induced negative effects on MVC.

### 4.4. Study Limitations

Several limitations should be acknowledged. Firstly, the sample was small and comprised young, recreationally active university students, which limits generalizability to athletic, older, or clinical populations; additionally, the sample-size calculation targeted ROM_max_, and recruitment was constrained by the limited availability of the SWE-equipped ultrasound system. Nevertheless, the shear *modulus* exhibited excellent reliability, and all the significant differences were above the MDC95.

No repeated assessments were performed during recovery; consequently, the duration of the observed changes and the time required for the shear *modulus* to return to baseline remain unknown; hence, whether the changes in the *modulus* were due to changes in intrinsic tissue properties after unloading cannot be exactly determined.

Maintaining the ankle at 80%ROM_max_ during SWE acquisition could have introduced additional passive loading that may have influenced the measured response. Although acquisition time was minimized and assessment procedures were standardized, the contribution of this measurement-related loading cannot be fully excluded.

A single muscle and two ankle positions and sites were sampled, thus limiting the generalization of the results to the whole plantarflexor complex. Moreover, joint angle and applied torque were not recorded during the stretching interventions. Therefore, equivalent mechanical intensity across protocols cannot be fully confirmed, limiting the extent to which differences in the observed responses can be attributed to stretching duration or loading pattern. Additionally, PS30 and PS45 differed in bout duration, cumulative time at the end ROM, and rest intervals, preventing the independent effects of these protocol components on the regional shear *modulus* from being exactly determined.

Finally, MVC was deliberately measured last in the sequence of data collection and may therefore have been influenced by the preceding measurements, although this ordering was held constant across conditions.

## 5. Conclusions

Acute stretching altered the passive mechanical behavior of the gastrocnemius medialis in a region- and muscle-length-dependent manner, with the pattern of response varying according to stretching duration and loading mode. At GM_mid_, PS30, PS45, and DS all reduced the shear *modulus* when the muscle was assessed in the lengthened position (80%ROM_max_), whereas no changes were detected at the neutral ankle angle. In contrast, the distal region adjacent to the MTJ showed a different response, with the shear *modulus* decreasing after PS45 and DS at both ankle positions, while PS30 produced no changes. These findings suggest that the distal GM may be particularly sensitive to the duration and/or mechanical pattern of the stretching stimulus. Overall, acute stretching seems not to induce a uniform mechanical response along the GM, depending on the muscle region examined, the characteristics of the stretching stimulus, and the muscle length at which local mechanical properties are assessed. These factors should therefore be considered when using SWE to evaluate or interpret stretching-induced changes in muscle stiffness. Longitudinal studies are needed to determine whether repeated exposure to different stretching protocols of different duration would produce sustained changes in regional GM mechanical behavior and whether these adaptations differ between stretching modalities.

## Figures and Tables

**Figure 1:**
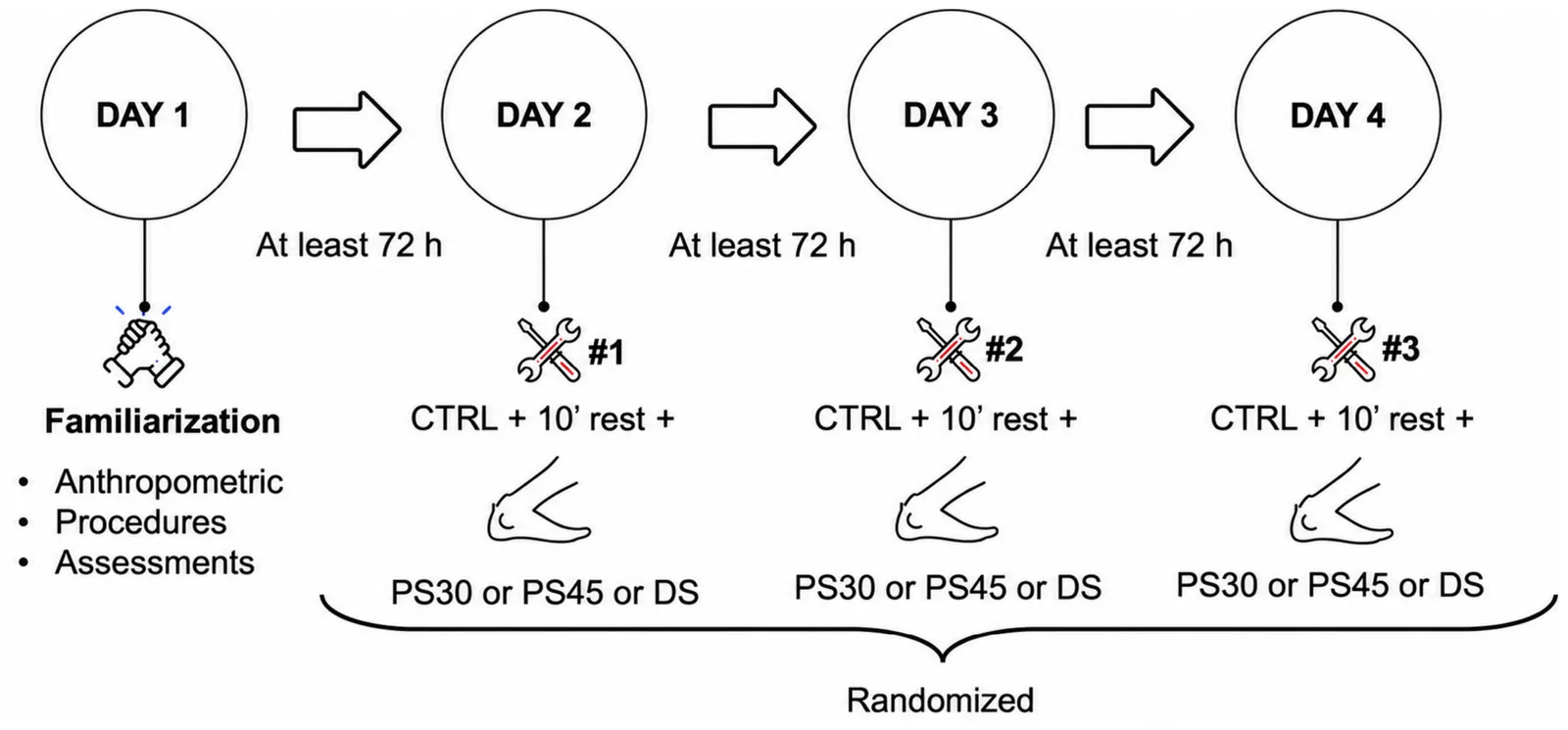
Experimental protocol. After familiarization on day #1, each intervention session was interspersed by at least 72 h of rest. The stretching protocols were randomized. Each intervention session was preceded by a control (CTRL) condition followed by 10 min of rest. PS30, five 30 s bouts of passive static stretching; PS45, five 45 s bouts of passive static stretching; DS, five sets of 30 dynamic stretching repetitions.

**Figure 2:**
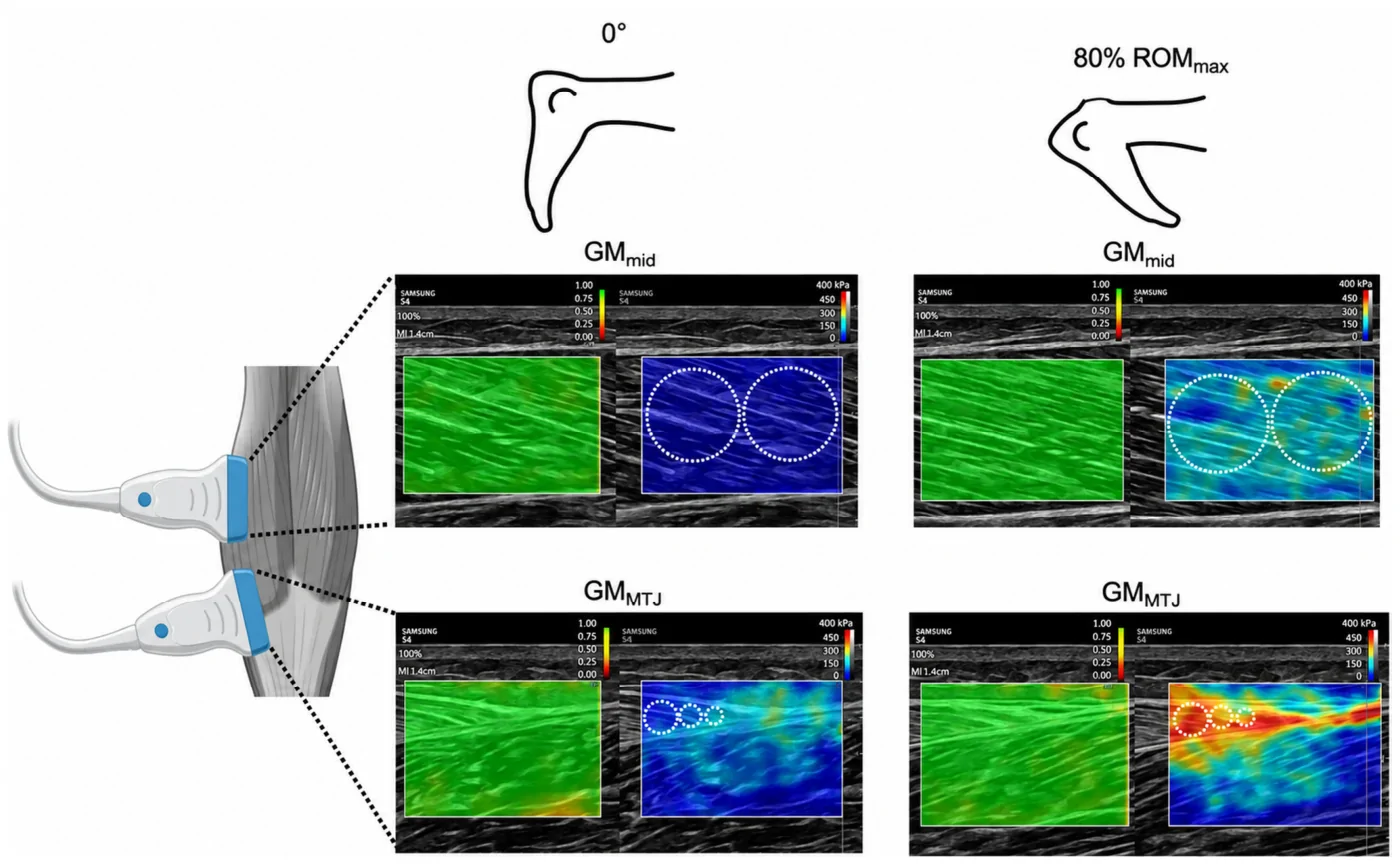
Representation of shear *modulus* determination at two ankle dorsiflexion angles. The mid-belly of the gastrocnemius medialis (GM_mid_) is shown on the upper panels, whereas the muscle part towards the GM myotendinous junction (GM_MTJ_) is shown on the lower panels. Each image displays two frames: on the left, reliability of the shear-wave signal is shown from dark brown (intraclass correlation coefficient − ICC = 0.00, not reliable at all) to bright green (ICC = 1.00 perfect reliability); on the right, the shear-wave elastogram is shown from dark blue (soft) to dark red (hard). The white dotted circles represent the region of interest within which the shear-wave *modulus* is automatically calculated and displayed. 0°, ankle in neutral position; 80%ROM_max_, dorsiflexion up to 80% of maximal range of motion.

**Figure 3:**
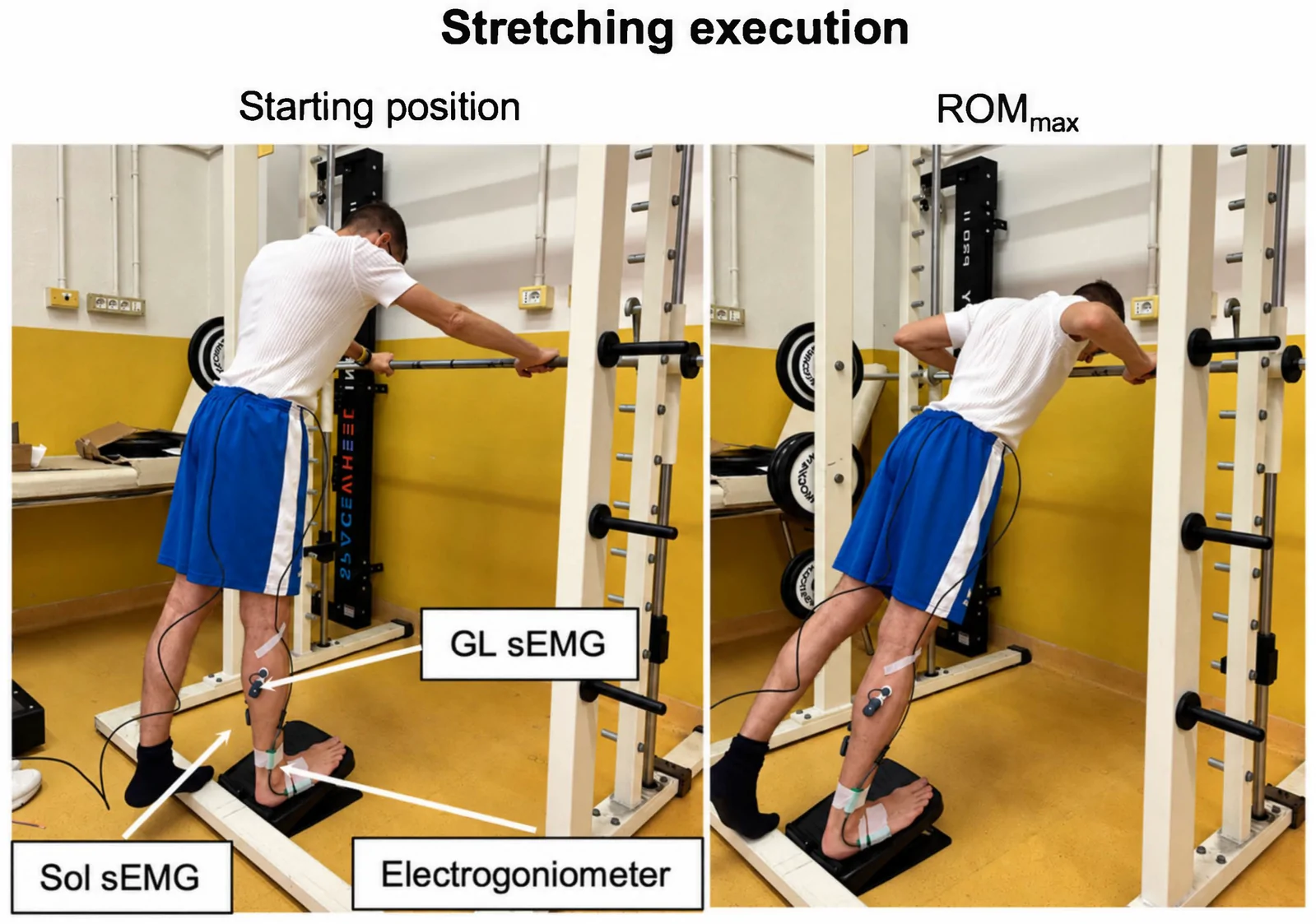
Example of stretching execution from the starting position (**left** image) to the maximal range of motion (ROM_max_, **right** image). See “Methods” for detailed description. sEMG, surface electromyography; GL, gastrocnemius lateralis; Sol, soleus muscle.

**Figure 4:**
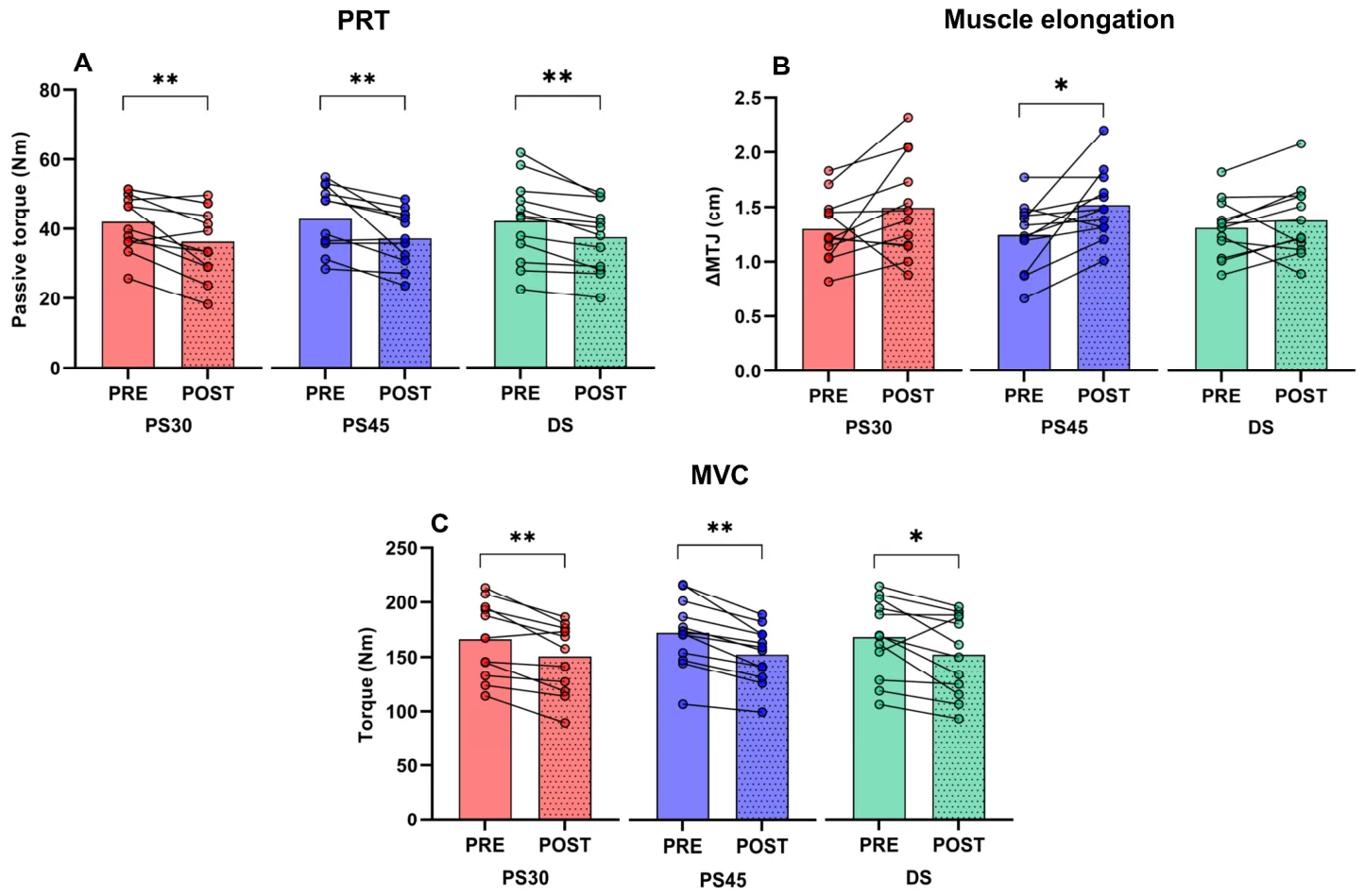
PRE- and POST-stretching absolute values for (**A**) passive resistive torque (PRT); (**B**) muscle elongation, assessed as myotendinous junction displacement (ΔMTJ); and (**C**) maximal voluntary contraction (MVC) torque. Bars represent group means; circles represent individual participants, with lines connecting PRE and POST values from the same participant within each protocol. PS30, five 30 s bouts of passive static stretching; PS45, five 45 s bouts of passive static stretching; DS, five sets of 30 dynamic stretching repetitions. PRE, before stretching; POST, after stretching. * *p* < 0.05; ** *p* < 0.01 for the corresponding within-protocol PRE–POST comparison.

**Figure 5:**
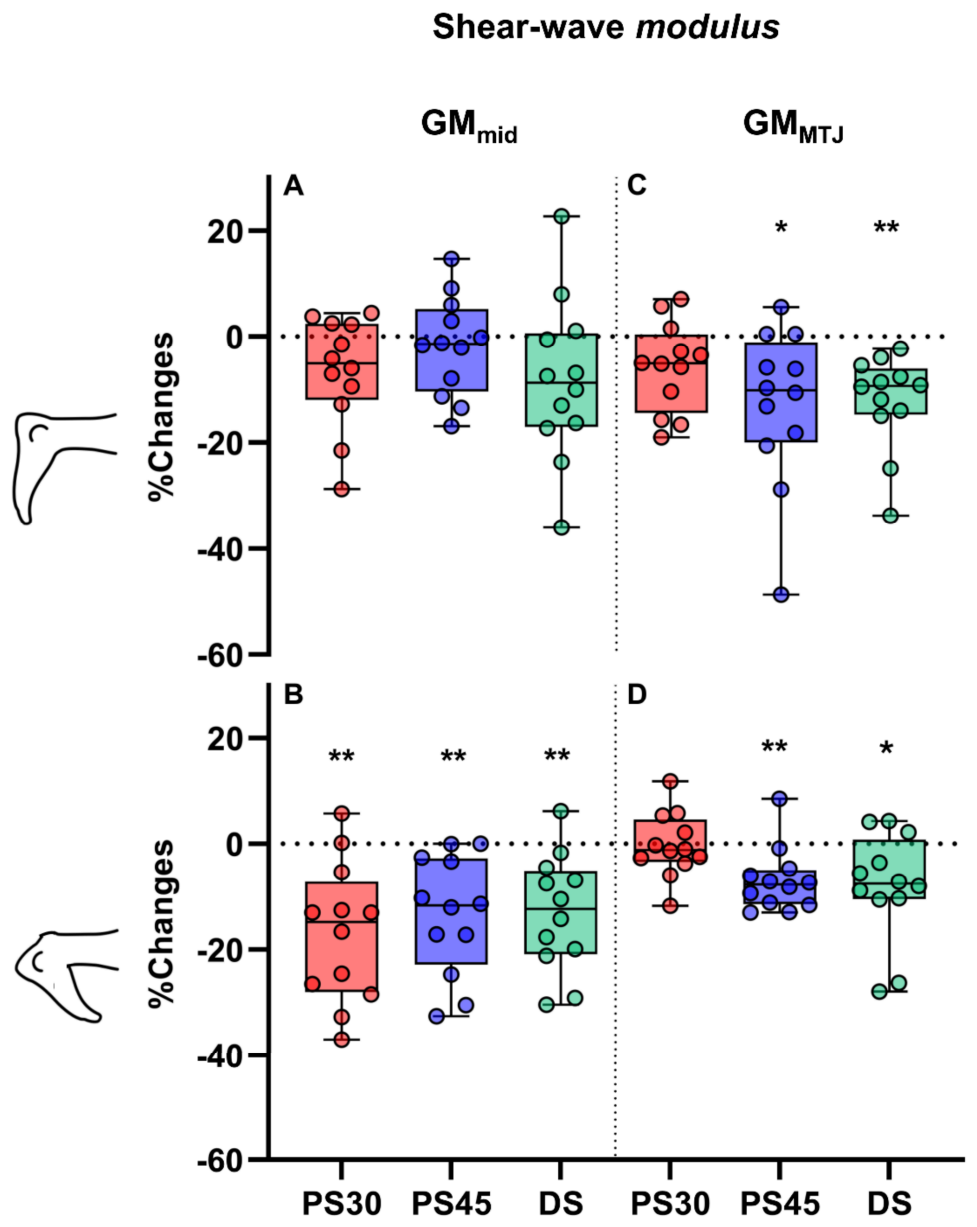
Stretch-induced percentage changes of the gastrocnemius medialis (GM) shear *modulus*. Panel A and B: Central portion of the GM muscle (GM_mid_) assessed at neutral ankle angle (**A**) and at 80% of maximal dorsiflexion (**B**). C and D: Muscular portion of the gastrocnemius medialis myotendinous junction (GM_MTJ_) assessed at neutral ankle angle (**C**) and at 80% of maximal dorsiflexion (**D**). PS30, five 30 s bouts of passive static stretching; PS45, five 45 s bouts of passive static stretching; DS, five sets of 30 dynamic stretching repetitions; *, significant change with *p* < 0.05; **, significant change with *p* < 0.01.

**Table 1:** Intra-day reliability of shear-wave elastography *modulus*.

	ICC (95%CI)	SEM (%)	MDC95 (%)
**GM_mid_ *modulus* at 0°**			
PS30	0.975 (0.914–0.993)	4.68	12.98
PS45	0.972 (0.906–0.992)	4.16	11.52
DS	0.967 (0.888–0.990)	4.01	11.11
**GM_mid_ *modulus* at 80%ROM_max_**			
PS30	0.921 (0.737–0.977)	2.75	7.62
PS45	0.970 (0.896–0.991)	2.17	6.01
DS	0.846 (0.452–0.956)	3.71	10.29
**GM_MTJ_ *modulus* at 0°**			
PS30	0.989 (0.963–0.997)	3.63	10.05
PS45	0.992 (0.972–0.998)	3.12	8.66
DS	0.990 (0.966–0.997)	2.95	8.18
**GM_MTJ_ *modulus* at 80%ROM_max_**			
PS30	0.985 (0.951–0.996)	3.02	8.38
PS45	0.994 (0.980–0.998)	2.26	6.25
DS	0.988 (0.959–0.997)	2.60	7.22

ICC, intraclass correlation coefficient; 95%CI, confidence interval at 95%; SEM, standard error of measurement; MDC95, minimum detectable change at 95% confidence as a percentage; ROM_max_, maximal range of motion; GM_mid_, central portion of the gastrocnemius medialis muscle; GM_MTJ_, muscular portion of the gastrocnemius medialis myotendinous junction; PS30, five 30 s bouts of passive static stretching; PS45, five 45 s bouts of passive static stretching; DS, five sets of 30 dynamic stretching repetitions.

**Table 2:** Comparison between each stretching session and its respective control session.

						ANOVA-RM	
PS30	PRE CTRL	POST CTRL	PRE PS30	POST PS30	Time	Condition	Time × Condition
ROM_max_ (°)	32.7 ± 3.6	32.6 ± 3.6	32.7 ± 3.0	35.3 ± 3.6 **	F = 7.39 ***p* = 0.02** η_p_^2^ = 0.40	F = 7.22 ***p* = 0.02** η_p_^2^ = 0.40	F = 28.1 ***p* < 0.001** η_p_^2^ = 0.72
GM_mid_ *modulus* at 0° (kPa)	42.60 ± 13.14	43.21 ± 12.27	43.03 ± 11.55	40.25 ± 12.63	Friedman test *p* = 0.06		
GM_mid_ *modulus* at 80%ROM_max_ (kPa)	227.05 ± 22.15	223.31 ± 21.91	230.86 ± 24.18	191.25 ± 35.00 **	F = 18.82 ***p* = 0.001** η_p_^2^ = 0.63	F = 9.51 ***p* = 0.01** η_p_^2^ = 0.46	F = 13.17 ***p* = 0.004** η_p_^2^ = 0.54
GM_MTJ_ *modulus* at 0° (kPa)	218.11 ± 73.98	221.19 ± 77.87	222.68 ± 79.18	208.86 ± 74.35	F = 2.25 *p* = 0.16 η_p_^2^ = 0.17	F = 0.55 *p* = 0.48 η_p_^2^ = 0.05	F = 3.41 *p* = 0.09 η_p_^2^ = 0.24
GM_MTJ_ *modulus* at 80%ROM_max_ (kPa)	406.22 ± 97.27	411.32 ± 104.48	408.81 ± 95.78	404.79 ± 87.38	Friedman test *p* = 0.44		
**PS45**	**PRE CTRL**	**POST CTRL**	**PRE PS45**	**POST PS45**			
ROM_max_ (°)	33.3 ± 3.1	33.4 ± 3.4	33.5 ± 2.9	36.1 ± 3.5 **	F = 29.1 ***p* < 0.001** η_p_^2^ = 0.73	F = 10.7 ***p* = 0.007** η_p_^2^ = 0.49	F = 48.1 ***p* < 0.001** η_p_^2^ = 0.81
GM_mid_ *modulus* at 0° (kPa)	40.72 ± 10.26	41.77 ± 10.23	40.95 ± 9.46	39.72 ± 6.94	Friedman test *p* = 0.38		
GM_mid_ *modulus* at 80%ROM_max_ (kPa)	227.62 ± 26.76	228.73 ± 30.38	226.38 ± 32.77	196.69 ± 41.89 **	F = 17.42 ***p* = 0.002** η_p_^2^ = 0.61	F = 14.39 ***p* = 0.003** η_p_^2^ = 0.57	F = 16.55 ***p* = 0.002** η_p_^2^ = 0.60
GM_MTJ_ *modulus* at 0° (kPa)	215.63 ± 75.24	213.79 ± 74.72	217.57 ± 75.34	191.58 ± 80.83 *	F = 10.85 ***p* = 0.007** η_p_^2^ = 0.50	F = 2.13 *p* = 0.17 η_p_^2^ = 0.16	F = 2.87 *p* = 0.12 η_p_^2^ = 0.21
GM_MTJ_ *modulus* at 80%ROM_max_ (kPa)	401.50 ± 116.91	398.49 ± 116.07	407.05 ± 112.96	379.48 ± 109.44	F = 13.30 ***p* = 0.004** η_p_^2^ = 0.55	F = 0.63 *p* = 0.44 η_p_^2^ = 0.05	F = 6.45 ***p* = 0.03** η_p_^2^ = 0.37
**DS**	**PRE CTRL**	**POST CTRL**	**PRE DS**	**POST DS**			
ROM_max_ (°)	33.8 ± 4.2	33.3 ± 4.1	33.3 ± 4.0	36.6 ± 4.0 **	F = 8.52 ***p* = 0.01** η_p_^2^ = 0.44	F = 23.43 ***p* < 0.001** η_p_^2^ = 0.68	F = 127.89 ***p* < 0.001** η_p_^2^ = 0.92
GM_mid_ *modulus* at 0° (kPa)	42.12 ± 8.93	42.90 ± 9.84	42.79 ± 10.05	38.19 ± 4.68	Friedman test *p* = 0.19		
GM_mid_ *modulus* at 80%ROM_max_ (kPa)	227.84 ± 22.87	229.10 ± 20.36	225.21 ± 26.04	195.67 ± 33.83 **	F = 14.27 ***p* = 0.003** η_p_^2^ = 0.56	F = 12.62 ***p* = 0.005** η_p_^2^ = 0.53	F = 10.76 ***p* = 0.007** η_p_^2^ = 0.49
GM_MTJ_ *modulus* at 0° (kPa)	222.88 ± 66.21	221.34 ± 64.80	226.56 ± 67.98	201.93 ± 70.84 **	F = 18.54 ***p* = 0.001** η_p_^2^ = 0.63	F = 3.41 *p* = 0.09 η_p_^2^ = 0.24	F = 19.55 ***p* = 0.001** η_p_^2^ = 0.64
GM_MTJ_ *modulus* at 80%ROM_max_ (kPa)	409.47 ± 99.43	410.12 ± 95.33	398.90 ± 109.41	366.72 ± 103.44	F = 5.87 ***p* = 0.03** η_p_^2^ = 0.35	F = 26.61 ***p* < 0.001** η_p_^2^ = 0.71	F = 4.45 ***p* = 0.05** η_p_^2^ = 0.29

Values are mean ± SD. PS30, five 30 s bouts of passive static stretching; PS45, five 45 s bouts of passive static stretching; DS, five sets of 30 dynamic stretching repetitions; CTRL, control session; ANOVA-RM, analysis of variance for repeated measures; ROM_max_, maximal range of motion; GM_mid_, central portion of the gastrocnemius medialis muscle; GM_MTJ_, muscular portion of the gastrocnemius medialis myotendinous junction; η_p_^2^, partial eta squared; *, significantly different from PRE with *p* < 0.05; **, significantly different from PRE with *p* < 0.01. Significant differences are highlighted in bold.

**Table 3:** Architectural parameters before (PRE) and after (POST) different stretching protocols at neutral ankle angle (0°) and at 80% of maximal dorsiflexion (80%ROM_max_).

Muscle Architecture	0°		80%ROM_max_				
PS30	PRE	POST	PRE	POST	Angle	Time	Angle × Time Interaction
L_f_ (cm)	5.66 ± 0.78	5.61 ± 0.86	7.36 ± 1.08 ^##^	7.30 ± 0.89 **	F = 186.51 ***p* < 0.001** η_p_^2^ = 0.94	F = 0.68 *p* = 0.43 η_p_^2^ = 0.06	F = 0.01 *p* = 0.92 η_p_^2^ = 0.001
θ (°)	21.60 ± 2.72	21.59 ± 2.96	17.49 ± 2.33 ^##^	17.44 ± 2.03 **	F = 53.45 ***p* < 0.001** η_p_^2^ = 0.83	F = 0.01 *p* = 0.92 η_p_^2^ = 0.001	F = 0.01 *p* = 0.93 η_p_^2^ = 0.001
MT (cm)	2.04 ± 0.20	2.02 ± 0.21	2.13 ± 0.21 ^##^	2.13 ± 0.18 **	F = 13.22 ***p* = 0.004** η_p_^2^ = 0.55	F = 0.19 *p* = 0.67 η_p_^2^ = 0.02	F = 0.22 *p* = 0.65 η_p_^2^ = 0.02
**PS45**	**PRE**	**POST**	**PRE**	**POST**			
L_f_ (cm)	5.73 ± 1.00	5.75 ± 0.98	7.01 ± 0.91 ^##^	7.00 ± 0.92 **	F = 145.40 ***p* < 0.001** η_p_^2^ = 0.93	F = 0.01 *p* = 0.94 η_p_^2^ = 0.001	F = 0.05 *p* = 0.82 η_p_^2^ = 0.01
θ (°)	21.23 ± 2.21	21.62 ± 2.09	17.65 ± 1.76 ^##^	17.52 ± 2.01 **	F = 102.97 ***p* < 0.001** η_p_^2^ = 0.90	F = 0.46 *p* = 0.51 η_p_^2^ = 0.04	F = 0.78 *p* = 0.40 η_p_^2^ = 0.07
MT (cm)	2.01 ± 0.24	2.01 ± 0.23	2.08 ± 0.20 ^##^	2.07 ± 0.19 **	F = 5.85 ***p* = 0.03** η_p_^2^ = 0.35	F = 0.33 *p* = 0.58 η_p_^2^ = 0.03	F = 1.65 *p* = 0.23 η_p_^2^ = 0.13
**DS30**	**PRE**	**POST**	**PRE**	**POST**			
L_f_ (cm)	5.79 ± 0.77	5.67 ± 0.78	6.96 ± 0.87 ^##^	6.95 ± 0.74 **	F = 255.94 ***p* < 0.001** η_p_^2^ = 0.96	F = 0.63 *p* = 0.45 η_p_^2^ = 0.05	F = 0.44 *p* = 0.52 η_p_^2^ = 0.04
θ (°)	21.32 ± 2.78	21.65 ± 2.43	17.73 ± 2.04 ^##^	17.91 ± 1.78 **	F = 133.11 ***p* < 0.001** η_p_^2^ = 0.92	F = 0.84 *p* = 0.38 η_p_^2^ = 0.07	F = 0.07 *p* = 0.80 η_p_^2^ = 0.01
MT (cm)	2.01 ± 0.23	2.01 ± 0.23	2.07 ± 0.20 ^##^	2.08 ± 0.21 **	F = 15.10 ***p* = 0.003** η_p_^2^ = 0.58	F = 0.82 *p* = 0.38 η_p_^2^ = 0.07	F = 0.64 *p* = 0.44 η_p_^2^ = 0.06

Values are mean ± SD. PS30, five 30 s bouts of passive static stretching; PS45, five 45 s bouts of passive static stretching; DS, five sets of 30 dynamic stretching repetitions; L_f_, fascicle length; θ, fascicle angle; MT, muscle thickness; ROM_max_, maximal range of motion; η_p_^2^, partial eta squared; ^##^, significantly different from PRE at 0° with *p* < 0.01; **, significantly different from POST at 0° with *p* < 0.01. Significant differences are highlighted in bold.

## Data Availability

The original data presented in this study are available from the corresponding author upon reasonable request.
